# PrEP in substance abuse treatment: a qualitative study of treatment provider perspectives

**DOI:** 10.1186/1747-597X-10-1

**Published:** 2015-01-08

**Authors:** Anya Y Spector, Robert H Remien, Susan Tross

**Affiliations:** HIV Center for Clinical and Behavioral Studies, Columbia University and NY State Psychiatric Institute, 722 West 168th Street, New York, 10032 NY USA

**Keywords:** PrEP, Substance abuse treatment, Providers’ perspectives, Integration of HIV prevention, Substance abuse treatment

## Abstract

**Objectives:**

To examine substance abuse treatment providers’ views on engaging clients in Pre-exposure Prophylaxis (PrEP) care and research trials.

**Methods:**

Thirty-six medical and counseling service providers in six New York City outpatient substance abuse treatment programs participated in semi-structured qualitative interviews. Thematic content analysis was conducted by three coders, independently.

**Results:**

Providers’ perspectives toward PrEP were characterized by six salient themes: 1) Limited PrEP awareness. 2) Ambivalence about PrEP; 3) Perception of multiple challenges to delivery; 4) Uncertainty about clients’ ability to be adherent to medication; 5) Concerns about medication safety/side effects; and 6) Perception of multiple barriers to conducting clinical trials.

**Conclusions:**

Despite anticipated challenges, providers supported the introduction of PrEP in outpatient substance abuse treatment. Comprehensive training for providers is needed and should include PrEP eligibility criteria, strategies to support adherence and medication monitoring guidelines. Linkages between substance abuse treatment and primary care and/or enhancement of capacity within clinics to offer PrEP may help facilitate PrEP delivery. When conducting research in outpatient clinics, it is particularly important to protect client confidentiality.

## Introduction

In the US, there are approximately two million substance users in community treatment programs who are at risk for HIV [1]. Because of injection drug use (IDU) and/or unprotected sex under the influence of drugs and/or alcohol, substance users, and their sexual partners, are vulnerable to acquiring HIV. Sexual transmission accounts for the greatest proportion of new HIV infections [2]. The association between sexual risk and substance abuse is well documented among opposite-sex, as well as same-sex partners ([3–5]).

Studies of New York City substance users in (D.C. [6]) and out of (D.C. [7, 8]) treatment for substance abuse show that injection drug users (IDUs) and non-IDUs have nearly the same prevalence of HIV due to the strong connection between sexual risk and substance abuse [9]. In particular, stimulants, especially cocaine [10], methamphetamine [11–13], “club drugs” [14] and alcohol ([15–17]) have been shown to be drivers of sexual risk behavior. Sometimes, this is in the context of “survival sex” or exchange of sex for money and/or drugs, which is also strongly associated with HIV risk ([18, 19]). Sex work, while criminalized and punishable by arrest and sometimes imprisonment, is common in New York City. While HIV infection among IDUs has markedly declined due to syringe exchange programs [20–22], IDU is still an important risk factor.

The interrelationship between HIV and substance abuse necessitates a coordinated effort for providers (e.g., counselors, nurses, physicians) of substance abuse treatment [23] to prevent HIV among this vulnerable population. There are several evidence-based HIV prevention interventions available, however they are often not integrated into substance abuse treatment practices. In HIV prevention, the gap between research and practice is known to be 15 – 20 years [24]. Pre-exposure prophylaxis (PrEP) has been shown to be efficacious in preventing HIV acquisition among individuals at risk for HIV infection, if they are adherent to its use [25], and it has recently been approved by the FDA. Thus far, PrEP initiatives have been focused on men who have sex with men (MSM) and HIV seronegative partners in serodiscordant couples [26]. As PrEP rolls out, it would be timely to conduct research to determine the feasibility, acceptability, and impact of PrEP on providers’ practices, in substance abuse treatment community programs.

While primary care providers in community clinics have expressed willingness to prescribe PrEP to patients that are at risk for HIV, they sometimes do not agree on how to determine PrEP eligibility. Likewise, they noted implementation challenges including: difficulties monitoring PrEP within the current primary care structure, managing adherence issues and side effects [27]. Physicians specializing in HIV primary care were more aware than generalists about PrEP efficacy and safety, yet most physicians expressed some concerns about prescribing guidelines and monetary costs for PrEP [28]. No study thus far has examined providers in non-primary care settings’ perspectives on PrEP, despite the likelihood that they too may be called upon to recommend PrEP to their patients/clients.

Substance abuse treatment providers are fundamental to the adoption and delivery of PrEP, and the implementation of PrEP research, through their roles in counseling and coordination of care with clients’ primary care providers. Yet, the personal and organizational motives and challenges that providers can anticipate -- in delivering PrEP and participating in PrEP research – are not known. Such information is crucial to developing PrEP programs in substance abuse treatment – that offer the greatest promise for integrating PrEP, or linkage to PrEP, within substance abuse treatment. Such information is also crucial to identifying potential approaches to engage substance users in PrEP, and monitor and support their adherence.

To fill these important gaps, we conducted a qualitative study of outpatient substance abuse treatment providers’ perspectives toward providing PrEP in their programs. This leveraged the existing platform of the Greater New York Node of the NIDA Clinical Trials Network (CTN) – a network of community-based treatment and medical care facilities dedicated to carrying out effectiveness trials of proven (pharmacologic and/or behavioral) interventions for substance users. Outpatient substance abuse treatment programs in New York City include psychosocial programs, methadone maintenance programs, and harm reduction programs that offer syringe exchange. In this study we included four programs that offer psychosocial treatment and two programs that offer both psychosocial and methadone maintenance. Three programs were hospital-based and three were community-based. In each of six programs, a complement of providers – including counselors, physician and Director – was recruited. Each provider participated in a semi-structured interview – which elicited his/her views of important issues for PrEP program and research implementation. In doing so, this study aimed to obtain critical information for the integration of PrEP into treatment. In particular, we aimed to: 1) inform best practices for implementing PrEP research in substance abuse treatment; 2) suggest strategies to optimize delivery of PrEP; and 3) identify needs for provider training and potential salient targets for training.

## Methods

### Recruitment

Eligible agencies offered: 1) outpatient substance abuse treatment services in New York City and 2) were in the Clinical Trials Network (CTN). Twelve agencies were eligible. Seven executive directors (EDs) were successfully contacted by the PI, first by introductory email, and then with a follow-up phone call. The PI explained the purpose of the study, inclusion criteria, and incentives. Further, inclusion criteria for participants required that six providers (one clinic director, one medical provider and four counselors) from each agency be interviewed. Each interviewee would receive a $40 cash incentive. Six EDs agreed to participate in the study. One ED declined to participate due to restructuring at the hospital where the program was based. All human subjects procedures were approved by the appropriate Institutional Review Board.

### Interview procedures

Using a community based participatory research approach (CBPR), which has been shown over the past two decades to enhance the external validity and relevance of research findings [29], the semi structured qualitative interview protocol was developed collaboratively over a series of discussions by the study’s PI (an experienced provider of both substance abuse and HIV services) and two community based collaborators: one with expertise in substance abuse treatment and one with expertise in HIV prevention. Interview development followed an iterative process to reflect the context of outpatient substance abuse treatment and the professional language commonly used in treatment settings so as to maintain a culturally appropriate stance.

The PI conducted interviews at each site in a private office space provided. The clinic director at each site scheduled interviews in advance so as not to disturb the workflow in the clinic and minimize burden on the clinic staff. Interviews lasted approximately 30 minutes and were audio recorded and transcribed verbatim. No names or any other identifying information were recorded. At the start of the interview, participants received a study information sheet, as well as a packet of information summarizing the “state of the art” evidence-based behavioral and bio-behavioral HIV prevention strategies and PrEP. Participants were guided through the information and were encouraged to ask questions about PrEP and to reflect upon what they had learned. Through open-ended questions, providers were asked to describe their reactions to PrEP, how they might anticipate implementing PrEP at their clinics and to what extent they would be receptive to conducting PrEP trials at their clinics. They were asked to describe barriers and promoters to these PrEP initiatives.

#### Sample characteristics

36 Providers (N = 36) were surveyed from 6 outpatient substance abuse treatment clinics (three hospital based and three community based). Two of the three hospital based programs and one of the Community based programs offered Methadone Maintenance. Eight providers were medical, six were clinic directors, and 22 were counselors.

The providers at the agencies held the following licenses/degrees: 10 held Masters of Social Work degrees, 5 held both Masters of Social Work degrees and Credentialed Alcohol and Substance Abuse Counselor (CASAC) designation, 1 had a Master of Social Work and a Master of Exercise Physiology degree, 2 held Bachelor’s degrees and CASAC, 2 held Bachelor’s degrees, 6 providers held only CASAC, one provider was a Licensed Practical Nurse, 1 provider was Registered Nurse, 1 provider was a Nurse Practitioner, 1 provider was a Physician Assistant, 5 providers were Medical Doctors, 1 provider held a Master of Science in Vocational Counseling.

### Qualitative analysis

Data analysis was conducted by the PI and the two community consultants, over a series of in person meetings, based on a modified Grounded Theory approach using thematic content analysis [30] and organized through ResearchWare HyperResearch qualitative data management software. Themes, and their variations, focused on providers’: (1) awareness of, and experiences with PrEP; (2) barriers and promoters associated with use of PrEP or with conduct of PrEP research with substance using clients (3) strategies for negotiating perceived challenges to participating in these initiatives.

The panel of three coders independently coded the first two interviews using a line-by-line open-coding strategy according to thematic content analysis to identify chunks of text that fall under the themes of inquiry, PrEP: 1) awareness/experiences; 2) barriers/promoters, and 3) strategies to overcome challenges and 4) providers’ perspectives, opinions, and practices related to PrEP. Meetings to compare coding and to achieve 100% consensus were held in order to build a codebook.

Subsequently coders coded the next two interviews and met to discuss and compare, adding additional domains as they emerged and meeting regularly until saturation occurred. Saturation of data occurred after twelve interviews. The codebook was finalized at this point. We then analyzed all transcripts based upon the codebook, which contained definitions of each domain. These definitions were used to mark the text of all transcripts. We marked only text that matched the definitions in the codebook. Once we determined which passages in the transcripts best represent the constructs in the codebook, a grid containing these passages was created. These passages (“quotes”) were then reviewed, and revised for grammatical clarity and entered into HyperResearch qualitative data management software.

Reports were extracted based upon the findings that emerged related to PrEP and organized according to content analysis. This resulted in the analysis of salient themes. Based upon the themes of inquiry, six areas emerged as most salient for the providers. These are: 1) Limited awareness of PrEP. 2) Ambivalent perspectives toward offering PrEP. 3) Challenges in delivering PrEP. 4) Uncertainty about adherence to PrEP. 5) Concerns about safety/side effects. 6) Barriers to conducting clinical trials.

## Results

Findings are presented across job roles, organizations, and educational backgrounds.

### Limited awareness of PrEP

There was an overall lack of awareness of PrEP in this sample, with only four of the 36 providers having had prior awareness of PrEP. One was a hospital administrator, a psychiatrist, who has had extensive involvement in clinical and behavioral research trials as a site co-PI with academic partners; one who was a psychiatrist who had recently completed a post-graduate research fellowship in addiction; one was a nurse practitioner who recently graduated with a Masters in nursing; and one was an MSW-level social worker who had recently completed graduated school. The remaining 32 providers said they had never heard of PrEP or were not aware that there were pharmacological approaches to HIV prevention. For 32 of the providers surveyed, the informational materials provided by the PI were the first instance they were learning of PrEP.

### Ambivalent perspectives toward offering PrEP

The four providers that already knew about PrEP felt favorably toward offering PrEP to their clients, stating that it would be beneficial to substance using clients, particularly because of sexual, and in some cases, needle-sharing risk. One medical provider mentioned that he had read “some studies” describing PrEP trials, however he was unaware that PrEP had since received FDA approval. Therefore, while this provider was aware of PrEP, the lack of awareness of PrEP’s availability would have prevented the provider from presenting this as an option to an at-risk client.

*“Yes. I’ve heard about -- research related to it. That you take high-risk people, like injection drug users, or people with high risk sexual behavior, and prophylactically give them antiretrovirals. And -- and my understanding of the research is that it’s highly effective in preventing, you know, similar to -- similar to giving antiretrovirals to mothers who were pregnant, you know, seriously reduced the risk of kids getting it” (M.D., hospital-based).*

For those providers who had not heard of PrEP, initial reactions to learning about PrEP were overwhelmingly positive. 29 providers stated that PrEP was a welcome advance in prevention and that it would be a benefit to their clients. All of the medical providers had a strongly positive reaction to PrEP and stated their support for offering PrEP to their clients. Counseling providers in general reacted favorably and considered PrEP as a tool that could be used as a complement to counseling.

*“I think if they’re already engaging in risky behaviors, I think it’s safe to be safer, and to have something in place, and not to just tell them not to do the risky behavior. You could work on the risky behaviors while they’re taking the pill and being safe at the same time. So then we could discuss how to discontinue risky behaviors but in the meantime you’re safe until they get to that point so I think that’s excellent.” (Counselor, community based).*

Four providers reacted negatively and 2 were neutral, mainly due to a belief in “risk compensation,” the notion that PrEP would result in a client feeling protected against HIV and therefore more likely to engage in risky sexual behavior [31]. These six providers (all counselors) believed in risk compensation and characterized PrEP as a “permission slip” to act out sexually. They suggested that PrEP would lead clients to engage in unprotected sex “recklessly” and objected to its use on this basis. They used a moral argument with regard to this medication stating that they had moral objections to offering it to clients.

*“I already know that everything is not for everybody, because we have some people that have very promiscuous behaviors. And I think a drug like this would probably or could -- has the potential to make them, like I said, more promiscuous.” (Counselor, community based).*

### Challenges in delivering PrEP

Providers had never implemented PrEP in their clinics, nor had they participated in PrEP research trials. Providers’ were asked about their *anticipated* practices for delivering PrEP in their outpatient substance abuse treatment setting. In other words, how they anticipate the process of implementation may look or how they suppose barriers to implementation may present.

Providers anticipated the following challenges regarding offering PrEP: 1) Educating clients through counseling about PrEP, when they themselves had very little/limited information about PrEP, 2) Prescribing and/or monitoring PrEP without qualified medical staff to prescribe (e.g., psychiatrists or physicians) and without sufficient funding for medication monitoring through blood tests, 3) determining eligibility criteria (i.e., how to identify which clients should receive PrEP), 4) making referrals to (primary care) providers who can prescribe PrEP without referral-making procedures and policies in place.

Nearly all of the counseling and some medical providers stated that their PrEP implementation would consist mainly of offering education and information to clients about PrEP as an option for HIV prevention. They were willing to discuss PrEP, hand out written materials, and offer general information about the availability of PrEP.

*“Right. Yeah. I would empower them, you know, with having, you know, that as a choice, you know, an option, you know, in their life, you know, it’s the same way I would -- might tell them, you know, about other programs and resources that are out there.” (Counselor, community based).*

However, in order to do so providers stated that they would need training about PrEP and how to present the information to clients.

*“Yeah, serious training. Just, basically, what to say, how to say it, and really something that we will remember as far as our own interpreting it into our own words and giving it to the client.”-counselor, community based.*

Despite willingness to offer information, only 7 said that they would make a referral for a client to obtain PrEP at a primary care clinic or in the case of hospital based programs that offered primary care, at primary care onsite services.

*“I think I would, you know, I would educate them about it. You know, I wouldn’t necessarily-- maybe I wouldn’t go ahead and make a direct referral to them….but I would raise it, you know, raise it to the client’s attention, you know, that it’s something, you know, that’s available, you know” (Counselor, community based).*

*“I’d just educate them. I wouldn’t recommend it. I would educate them that it’s out there, and that’s it.” (Counselor, hospital based).*

*“Yeah, we have to offer the alternatives. Now, I’m not endorsing it. That’s the caveat. I’m not endorsing it, but these are your options. This is what’s out there, you know, and please speak to your physician.” (Counselor, hospital based).*

A few medical providers were inclined to make referrals and to help the client obtain PrEP. Being situated in a hospital setting seemed to encourage referral making, especially for primary care and HIV antibody testing because of the natural link between the substance abuse program and the larger health care system.

*“Well, we’re lucky, in a sense, that we can -- we do have, like, a very, very strong sort of medically-based presence here in this treatment program. Many programs do not have that luxury. But then, we also are, you know, in a hospital. We can have a relationship with the virology clinic”-(Medical provider, hospital based).*

The program directors shared the following concerns: 1) that they did not have prescribers and/or sufficient staff available, 2) did not have the capacity to monitor PrEP adherence, and 3) were unsure about how PrEP would be covered in terms of third-party reimbursement.

*“I think what you’re going to hear from many of the staff is that they feel like it’s just one more thing on my plate. They feel like they have so much work to do because, as I mentioned,-- our staffing has gone down but the amount of work that we do has stayed the same. So you basically have fewer people doing more work. You know, at least in our clinic, in our setting -- and I know other settings have been different, but we’d never had productivity requirements. Now all the providers have productivity requirements.”* (Program Director, hospital based).

Two program directors reported that their staff would need comprehensive training on PrEP in order to be able to educate clients. Further, one program director noted that staff needed training in psychopharmacology in general because many clients are dually-diagnosed with mental health conditions and require counseling around medication adherence for other disorders and conditions.

*“So, I think that that would depend -- they would require a lot of education, because I think our staff, in general… Well, I would say the younger staff is more open, and they have more education. We do have some older staff who have been in the field for a long time, maybe don’t have as much education, and tend to shy away from medications, in general. So, even to get them to understand, like, a co-occurring disorder, and a client would benefit from taking a psychotropic medication of some sort along with their treatment regimen -- is a good thing. It -- that requires a lot of education.” (Program Director, community based).*

Overall providers’ criteria for clients to receive PrEP was two-fold: exhibiting a demonstrated risk either through exchange sex, unprotected sex with multiple partners, or needle sharing; and exhibiting psychosocial stability demonstrated by stable housing, program attendance, sobriety, medication compliance and overall medical health. When asked what types of clients would be good candidates for PrEP, 22 providers said that the client’s level of risk should guide the assessment of whether they are offered PrEP.

*“So if you look at something like that and you take an assessment of how -- what risk level they’re at, then I would suggest absolutely, why not put the medication in there. But I think there should be a -- there should definitely be a risk level and -- yeah.” (Counselor, community based).*

When asked to elaborate they used the following terms to describe such clients: “high risk”, “risky behaviors”, “don’t want to use condoms” or “multiple partners” and “promiscuous” to describe the ideal candidate for PrEP. Two providers stated that PrEP would be ideal for “prostitutes” and three mentioned IDUs.

*“OK, well, a client that -- let’s say a client that has reported a sex addiction, and has multiple partners, doesn’t wear a condom, things of that nature. Clients such as that I would probably recommend that to.” (Counselor community based).*

*“Or just, you know, like I said, people who are high risk because maybe they are promiscuous or maybe they are interested in, you know, using prostitutes or because they are still sharing needles” (Counselor, hospital based).*

Eight providers stated that PrEP would be ideal for serodiscordant couples and one counselor described PrEP as a way for his HIV positive client to have “normal marital relations”. Providers appeared most comfortable and approving when discussing using PrEP for serodiscordant couples. One provider explicitly stated that that takes the “moral question” out of it. Two providers mentioned young age as a criterion, stating that they felt that younger clients (under age 30) are most at risk due to having greater numbers of sexual partners and being generally more sexually active, whereas the older clients were engaged in stable and presumably monogamous partnerships.

*“You know, young people who are sleeping around -- I mean, you know, that would be a great thing to take. You could prevent it -- if you can’t convince them to wear a condom, you could take this.” (Psychiatrist, hospital based).*

Four providers stated that being “stable” medically and having achieved a minimum length of time (between three and six months) of abstinence from drugs were requisites, as well as being compliant with their other medication regimens (e.g., psychotropic medications). One provider mentioned stable housing as a requirement. One director of a program mentioned that regular attendance and not missing appointments were important criteria.

*“it’s counterintuitive, because the client who’s going to be a client that would benefit from this is likely to be a client who would be least likely to comply with all the stipulations that would be associated with it…Yeah, the two things would go hand in hand, because the person who’s running around having a lot of risky behaviors is generally not a person who’s going to a primary-care doctor, and not a person… You know”- Program Director, community based.*

*“If you know your patient has history of multiple partners, and a history of being homeless, and a history of selling her body, and a history of constantly using drugs, then you may be hesitant on giving this patient this pill. You know what I mean? But, you may say here, you need it. So you’ve got to weigh your pros and cons, and every patient’s different. Their social -- you know, their social skills, their living situation, everything you’ve got to take into account”.- medical provider, hospital based.*

### Uncertainty about adherence to PrEP

Seventeen providers comprised of both counselors and medical providers, reported that adherence would be a serious concern for their clients. They stated that their clients’ continued drug use, unstable housing, or simply forgetting to take the medication may impinge upon clients’ ultimate ability to maintain compliance of daily dosing. Uncertainty about adherence gave some providers pause when considering whether they would recommend PrEP, particularly in light of the potential consequences of skipping doses (e.g., developing resistance). One psychiatrist asked, “How long does it last?” in an effort to establish the length of time that a client would experience a prophylactic effect even if they were to miss a dose.

Most of the medical staff reported that they were concerned about non-compliance with this population.

*“They would definitely need to be monitored, I’m sure. With these kind of clients they need consistency. So they’re not going to do it on their own. They’re not going to remember.”- (Medical provider, community based).*

Five non-medical providers reported that adherence would not be a concern and that their clients would indeed be able to comply with medication, particularly because many clients already take medication for medical or mental health conditions.

*“..you have outpatient, then you have the methadone clients. Those guys I don’t think would have a problem [with adherence]. – (Counselor, hospital based).*

### Concerns about PrEP safety/side effects

The majority of providers relayed concerns about long-term effects of PrEP and safety for clients that have co-morbid medical issues (e.g., hepatitis C) some asked about how PrEP may interact with other medications that clients were taking (e.g., methadone). One provider asked about PrEP’s safety during pregnancy, and one provider asked about whether PrEP had been tested with adolescents. Most providers, both medical and non-medical, were reluctant to offer or recommend PrEP to clients that were already ill with chronic conditions because of concerns about liver toxicity.

*“Right. OK. Yeah. I think definitely, because you want to find out in the event that, you know, is it safe to take with methadol. Is it take to -- take with suboxone, is it safe to, actually, take with any other medications they may be taking for their medical issues.”- (Counselor, hospital based).*

Concerns over side effects were less prevalent than safety concerns, although one provider pointed out that if the client experiences side effects he/she may discontinue the medication therefore it is important to consider the severity of the potential side effects.

### Barriers to conducting clinical trials

Most providers agreed that participating in a trial of PrEP would be a benefit to their clients because they would be able to secure access to the medication for no cost. Nearly all of the providers stated that they would be willing to be involved with a PrEP trial in terms of conducting study tasks and procedures (e.g., recruitment, data collection).

Three main areas of concern emerged. 1) Informed consent. Providers affirmed necessity of clients understanding the risks and benefits prior to engaging in PrEP research. They stated that clients would need to be well counseled around drug safety and potential side effects, particularly because many clients have a low education level and may be compromised cognitively due to drug use. 2) Maintaining confidentiality. Seven providers were concerned about securing confidentiality in offering PrEP on site through clinical trials. They were hesitant to engage clients in trials if there was a risk of clients being stigmatized by peers or staff due to being perceived as “at risk” or as part of a serodiscordant couple. Moreover, concerns about maintaining the confidentiality of clients’ partners or significant others arose because peers may conclude HIV status of partners if the client is enrolled in a PrEP trial.

*“But will people will come saying, you know, I have a partner or I have, you know. And then, at that point, does the HIV status of the partner become identified? Hopefully not. That’s not what we’re interested in. We’re interested in protecting the patient.”- (Medical provider, hospital based).*

3) Mistrust toward research. Five counseling providers mentioned mistrust toward research as a potential barrier to engaging clients in PrEP trials. Clients may be reluctant to take PrEP and/or enroll in trials for fear of being treated as “guinea pigs”.

*“I don’t know. I think people like, just medication in general, they think of the side effects, they think of testing as being used as guinea pigs, as research work as guinea pigs, and what if there is something along the way that comes up?”- (*Counselor, community based).

Providers related this perception to historical abuses of vulnerable populations and people of color in general by the scientific community, as well as to conspiracy theory beliefs about HIV.

4) Role of incentives in recruitment. Six providers advocated monetary incentives as the most important strategy that may help overcome barriers to enrolling clients in PrEP trials.

*If the incentives were good, definitely a lot of people are open to research trials*. – (Counselor, community based).

#### Limitations

One limitation of our study was the selective nature of the sample as being nominated by the program directors for the interviews. However, despite being hand-selected by the administrators, the providers interviewed were not exceptionally knowledgeable about HIV, nor particularly experienced with HIV prevention. Therefore, we believe that the providers interviewed are adequate representatives of their respective organizations. Thus, the same themes and issues emerged across clinic and across job role from our data suggesting that this population has many common concerns and practices. The main limitation was that the clinics in this study might not represent all substance use treatment programs in the U.S. or in New York. The clinics we interviewed are relatively well resourced and participate in the Clinical Trials Network (CTN), therefore may be more receptive to research than non-CTN affiliated clinics.

## Discussion

To our knowledge, this is the first study to explore substance abuse treatment providers’ views on PrEP. Given the HIV risk faced by many substance-using clients, it is crucial to assess the potential for implementation of PrEP within substance abuse treatment settings and to understand the perspectives, barriers and promoters from the standpoint of the providers that will ultimately be responsible for PrEP’s uptake in these settings. A few major themes emerged from our study of medical staff, counseling providers and directors. These findings cut across the various job roles and clinic settings.

Only a small proportion of our sample (approximately 10%) had ever heard of PrEP prior to the interview. This was surprising, given the fact that the clinics are located in New York City, the epicenter of the HIV epidemic and boasting cutting-edge HIV research and services. The overwhelmingly positive response of most providers upon first learning of PrEP was encouraging given the controversy surrounding PrEP, and suggests that this work force is open to innovation and to using pharmacological interventions. This is not surprising given the fact that many work with clients that are prescribed psychotropic medications and opiate-substitution regimens. The providers who voiced objections to PrEP did so mostly on the basis of risk compensation, which is an argument that has, in the past, been used to argue against oral contraception, condoms, etc. Risk compensation has been shown in some populations (e.g., men who have sex with men) with regard to inconsistent condom use leading to riskier sex with multiple casual partners, however, there is yet to be any empirical support for risk compensation among those receiving PrEP [32].

Behavioral counseling has been recommended as a strategy to use in concert with pharmacological interventions like PrEP in order to increase clients’ risk perception and to mitigate the potential consequences of risk compensation [33]. Thus counseling providers may play a crucial role in reducing risk compensation if this effect is indeed found among PrEP recipients. Despite not having heard of PrEP providers were receptive to learning about PrEP and agreed that PrEP could be beneficial to their clients. Therefore, opportunities exist to enhance providers’ understanding of PrEP by targeted education. Future research may seek to identify dissemination channels that are aligned with providers’ preferences to circumvent barriers to training such as lack of time or funding.

Providers perceived their roles in PrEP implementation in different ways. Because our sample consisted mostly of counselors (78%) that do not prescribe medication it is not surprising that counselors considered their primary role to offer clients PrEP “information and education”. However, even medical providers in our sample that could prescribe PrEP, declined to do so because their roles in the clinics were not to provide primary care but rather, psychiatric care (e.g., antidepressants) or care related directly to substance use (e.g., buprenorphine, methadone). Nearly all providers, medical and counseling, were willing to offer information or education to clients (e.g., pamphlets). While this is an important initial step toward engaging clients in PrEP, it is equally important to help clients gain access to PrEP through formal referrals to obtain the medication, and only 20% of our sample including medical and non-medical providers, stated that they would be willing to offer referrals for obtaining PrEP. While most providers stated that they felt PrEP would be a benefit to their clients, their lack of willingness to make referrals indicates ambivalence toward PrEP and/or perhaps, toward making referrals. Future research needs to develop and test strategies to overcome barriers to referral making among providers of substance abuse services. Likewise, developing strategies to foster linkages between substance abuse treatment medical staff, counseling staff, and primary care services may help to facilitate referrals for PrEP.

The program directors’ concerns about implementation were related to three programmatic issues: 1) lack of medical staff to prescribe and monitor PrEP, 2) cost of PrEP and third party reimbursement (i.e., Medicaid), and 3) securing training for providers on how to educate clients about PrEP. Policymakers interested in the roll-out of PrEP within community based organizations including substance use treatment programs need approaches that take into account issues of cost, capacity and resource allocation so that PrEP treatment can be sustained and managed within these settings. Since most clients receiving treatment services in the locations surveyed are Medicaid recipients, it is important to advocate for Medicaid’s continued coverage of PrEP. Likewise, policymakers may include outcome measures on PrEP uptake for the purposes of reporting program-level data to funders and licensing agencies in order to motivate the use of PrEP as well as develop buy-in among implementers at the program level.

Most providers deemed clients that engage in “high risk” sexual behavior (e.g., unprotected sex, multiple partners), sex workers, clients that inject drugs, and serodiscordant couples as potential candidates for PrEP. However, they also considered stability in housing, mental health, abstinence from illicit drug use, and medical wellness as important criteria. Thus, the very same individuals that are most in need of PrEP according to providers may not be eligible for PrEP based upon the aforementioned criteria. The conflict inherent in targeting PrEP to those that are most chaotic and at-risk while limiting eligibility to individuals that are most stable points to an area of confusion. Providers need guidelines for identifying appropriate substance using clients in community settings so that they may conduct initial screening for PrEP prior to initiating a referral and/or offering to prescribe PrEP. Relatedly, most providers were concerned about adherence to a daily medication for this population in particular and had doubts about clients’ ability to comply with dosing due to forgetting, relapsing with drugs/alcohol, or due to a history of non-compliance with other medications.

Currently in development, long-acting injectable PrEP has been shown to be effective in monkeys and will soon be tested in humans [34]. Future research investigating other forms of PrEP for substance users such as long-acting injectables and vaginal rings may help minimize adherence concerns. Developing methods of delivery other than daily oral dosing may avert issues of compliance, maintaining abstinence from drugs, and reduce reliance on access to resources like housing or food, which may impede clients from taking oral PrEP. Therefore, alternative forms of PrEP may be most responsive to the lived experiences and real-world challenges experienced by substance users. In the meantime adherence interventions may be used to foster compliance with medication regimens [35].

Most pronounced were providers’ concerns about drug safety and side effects, particularly in light of the medically compromised clients that are already infected with HCV. Long-term effects of taking antiretrovirals were also noted and providers wanted to know whether it was known how clients’ livers and other organs would be affected over time. Based upon the providers’ focus on this area of concern, we hypothesize that any ambivalence toward PrEP was rooted in being unconvinced of PrEP’s safety and a fear of harming their clients. Thus, training for providers targeting these issues may help to overcome potential barriers to referring clients for PrEP.

However, it is worth noting that concerns about confidentiality raise the issue of PrEP stigma and HIV stigma with this population. The concern that clients might identify peers as PrEP recipients, should PrEP trials be conducted on site, and that this may result in stigmatization, points to a need to protect clients’ confidentialities, while at the same time working to educate the substance abuse treatment community about HIV, PrEP, and striving to dispel myths about HIV transmission, reduce homophobia, and assumptions about what constitutes risk.

Providers’ overwhelming support of conducting PrEP research trials on site and engaging their clients in trials may be an artifact of the sampling frame being CTN-affiliated programs. All of the programs are currently, or have in the past participated in clinical, behavioral and in many cases, pharmacological research through the CTN. Therefore, these programs in particular have a positive orientation toward research collaboration so this may not represent all substance use treatment programs in New York City or in the United States. This study suggests that more research ought to be done prior to rolling out PrEP in substance abuse treatment programs. PrEP research should focus on testing implementation strategies include structural interventions in order to build agency capacity to sustain PrEP. Interventions aimed at enhancing linkages to PrEP prescribers ought to be leveraged for those programs that lack the capacity to do so.

## Conclusion

The workforce of substance abuse treatment providers is poised to engage their at-risk clients in PrEP through counseling, referrals, research trials, and may promote sustained use of PrEP by helping clients to maintain adherence to medication regimens through counseling and monitoring. Policymakers and funders will play a critical roll in establishing the structural supports for these initiatives. Structural supports may include trainings for providers in a variety of HIV prevention strategies including PrEP. Trainings may be delivered in-person, through webinars, or through short films depending on budgetary constraints and technological capacity. Likewise, clinics that provide substance abuse treatment services may form collaborative partnerships with primary care providers that are equipped to prescribe PrEP. Such partnerships may be cultivated through policy initiatives aimed at integrating medical and behavioral services, a goal of the Affordable Care Act [36]. This study helped to fill a gap in the literature surrounding substance use treatment providers’ perspectives toward the roll-out of PrEP as a new tool for HIV prevention, their willingness to engage their clients in PrEP research, and the challenges that they anticipate to delivering PrEP to their population. Despite many challenges including limited awareness, misconceptions about PrEP, concerns about safety, and lack of capacity to prescribe and monitor PrEP, providers were highly motivated to learn more through training, and were deeply committed to helping their clients stay healthy. Given access to training, supervision, and linkages to appropriate referral sources, substance abuse treatment providers are likely to engage at-risk clients in accessing PrEP.
